# A qualitative process evaluation of a randomised controlled trial of a parenting intervention in community (school) settings for children at risk of attention deficit hyperactivity disorder (ADHD)

**DOI:** 10.1186/s12888-015-0670-z

**Published:** 2015-11-17

**Authors:** John A. Taylor, Althea Z. Valentine, Edward Sellman, Kate Bransby-Adams, David Daley, Kapil Sayal

**Affiliations:** University of Nottingham, NIHR Nottingham Hearing Biomedical Research Unit, Ropewalk House, 113 The Ropewalk, Nottingham, NG1 5DU UK; University of Nottingham, C Floor, Institute of Mental Health, UNIP, Triumph Road, Nottingham, NG7 2TU UK; University of Nottingham, Room B15, Dearing Building, Jubilee Campus, Wollaton Road, Nottingham, NG8 1BB UK; NIHR Clinical Research Network: East Midlands, D Floor, Institute of Mental Health, UNIP, Triumph Road, Nottingham, NG7 2TU UK; School of Medicine, University of Nottingham, & CANDAL (Centre for ADHD and Neuro-developmental Disorders across the Lifespan), Institute of Mental Health, UNIP, Triumph Road, Nottingham, NG7 2TU UK

**Keywords:** Parenting intervention, Recruitment, Retention, Barriers, School, ADHD, Qualitative evaluation, PATCHWORK

## Abstract

**Background:**

Interventions for parents of children experiencing emotional and/or behavioural difficulties can help to improve their children’s health, educational and social outcomes. However, the desirability and acceptability of screening and offering such interventions for attention-deficit hyperactivity disorder (ADHD)-type problems are currently unclear. This article is a qualitative process evaluation of a pragmatic cluster randomised controlled trial (Trial registration: ISRCTN87634685; reported elsewhere) to assess the feasibility and acceptability of a school-based parenting intervention programme for parents and teachers of children with high levels of ADHD symptoms.

**Methods:**

Parents (*n* = 22) and teaching staff (*n* = 29) took part in semi-structured group or individual interviews, either by telephone or face-to-face, following the main trial. Interviews were digitally-recorded, transcribed verbatim and subjected to thematic analysis.

**Results:**

The parenting intervention was acceptable to parents and teachers, and they were enthusiastic about the need for parenting groups in the school environment and stressed the importance of parent-school collaboration. Parents generally stated a preference for universal recruitment approaches to such programmes whilst teachers described the need to target specific parents.

Most parents who took part in the parenting intervention described it favourably and many saw benefits, at least in the short-term. Parents differed in their preferred group size, with some desiring one-to-one sessions and others favouring a larger group. Non-attending parents reported barriers to attendance such as fear of attending in a group, previous use of the programme, work and other commitments. Suggestions to improve the programme included: clearer communication; offering booster sessions; and greater collaboration with teachers.

**Conclusions:**

It is feasible to deliver parenting intervention programmes within or near schools. The intervention was acceptable to the majority of parents, thus retention was high, but recruitment was difficult and reaching the parents with the most need was challenging. The findings of the process evaluation identified greater benefits to families than were apparent in the main trial. Recommendations identified by parents and teaching staff may be used to inform service delivery and future research to enhance recruitment to parenting interventions in the school environment.

## Background

### Attention Deficit Hyperactivity Disorder (ADHD)

Attention Deficit Hyperactivity Disorder (ADHD) affects up to 3–5 % of school-aged children [1] and is characterised by pervasive and developmentally inappropriate levels of inattention, hyperactivity and/or impulsiveness which result in impairment. It is associated with a wide range of adverse outcomes, including increased risk of mental health difficulties, antisocial behaviour, and educational difficulties [2]. A stepped care approach for the identification and management of children’s challenging behaviour is recommended in the National Institute for Health and Clinical Excellence (NICE) guidelines for ADHD [1]. The guidelines suggest that behavioural intervention programmes for parents of children exhibiting high levels of ADHD-type difficulties may be beneficial.

### Recruitment to parenting interventions

There is evidence from systematic reviews that early intervention parent programmes are effective for improving a variety of outcomes including reduced parental depression and stress, and improved behaviour in children [3, 4]. Although Randomised Controlled Trials (RCTs) have demonstrated the efficacy of parenting interventions, in ‘real world’ settings numerous factors influence the success of such programmes, in particular recruitment and retention/engagement. In terms of recruitment, uptake to parenting interventions is variable, although some clinical studies have found most parents offered a place take it up [5, 6]. However, from those who do take part, drop-out rates can be high, particularly for parents with children with ADHD [7]. A recommended next step in parenting programme research is to explore non-engagement and barriers to attendance, as these are vital to the success of parenting interventions in community settings [8, 9].

Recent review articles have aimed to identify factors associated with attendance at parenting intervention programmes. In a qualitative synthesis of parents’ and professionals’ perceptions of such programmes [10], the authors described situational barriers (e.g. transport, childcare, inconvenient timing/venue) and psychological barriers (e.g. fears/worries, stigma, distrust), with dropout reflecting dislike of group activities, perceiving the programme to be unhelpful, problems implementing the strategies and changes in circumstances. Facilitators to attendance included effective advertising, direct recruitment, the programme being perceived as meeting families’ needs and the qualities of the therapist providing the training. In another review [11], the authors also highlighted perceptual barriers (e.g. programmes being intrusive, not relevant or too demanding) and programme factors (e.g. course content, styles of delivery). A qualitative systematic review [12] examined parents’ perceptions relating to the benefits gained through attendance and preconceptions about expectations. Parents reported that the skills and insights gained from the programme, together with feelings of mutual support from other attendees, helped them to re-establish control and gain confidence in their parenting, reducing their earlier feelings of inadequacy.

Kazdin & Wassell [13] found that higher levels of parent psychopathology and lower levels of quality of life predicted parental perceived barriers to treatment and therapeutic change in children referred for oppositional, aggressive and antisocial behaviour. The relevance (e.g. focusing on problems that parents perceive as difficult) and demands (e.g. too long, too confusing) of treatment, as perceived by parents, were significantly related to therapeutic change. A recent study which considered parents’ and practitioners’ views of parenting interventions for families living with ADHD found that many barriers mirrored those identified in more generic parenting intervention research, but also highlighted a number of ADHD-specific themes [14]. In particular, Smith and colleagues highlighted that parenting interventions for families with a child with ADHD need to consider the needs of the parents (e.g. self confidence, parental ADHD, depression) as well as the needs of the child and the initial approach to families needs to be tailored. Parental motivation to change parenting practice was noted as influencing both accessing/engaging in parenting interventions and the treatment effectiveness; the authors acknowledged that this may be because motivational deficits have been found in adults with ADHD [14].

Although the aforementioned research [14] has looked at parenting interventions in families living with ADHD, this study mainly considered pre-school aged children. There is a paucity of research evaluating the implementation of preventative parenting interventions for children with symptoms of ADHD in the school environment. Indeed, some research suggests that schools do not perceive parenting interventions as a solution to behavioural problems [15]. Hence there is a need for research to explore this, particularly for children exhibiting inattention/hyperactivity. Children exhibiting ADHD-type difficulties may present many challenges in contemporary school-based settings, which demand a high degree of control and focused attention and present challenges to classroom management for teachers. There is therefore also a need to obtain views from school staff about the feasibility and desirability of providing parenting interventions in the school environment.

### Main trial and process evaluation

In order to address these gaps in the literature, the PArents, Teachers and CHildren WORKing Together (PATCHWORK) pragmatic cluster RCT was conducted to evaluate the effectiveness and cost-effectiveness of a ‘1-2-3 Magic’-based parenting intervention for parents of 4–8 year olds in UK primary schools [16]. ‘1-2-3 Magic’ is a behavioural management programme for parents [17] and has components specific to ADHD. The PATCHWORK RCT aims to assess the acceptability and feasibility of offering a parenting intervention to parents of children with high levels of hyperactivity/inattention. This study is a unique implementation study as parents of children with high levels of hyperactivity/inattention were identified through a universal screening process, rather than targeting a help-seeking population. Participants therefore reflect a community-based sample of parents who are not necessarily seeking help, and where children do not necessarily have diagnosed difficulties.

This article reports the findings from a qualitative process evaluation of the PATCHWORK trial, which sought views about participants’ experience of all aspects of the study from screening to completion. Process evaluations examine the implementation of research and enable a better understanding of how an intervention is implemented and received within its specific context, thus aiding the interpretation of its outcomes [18, 19]. The research question sought to understand parents’ and teachers’ perceptions of the feasibility and acceptability of delivering a group-based parenting intervention in a school setting for parents of children with high levels of hyperactivity/inattention. Views were obtained from parents who attended the programme (attending parents) and parents of children with high levels of hyperactivity/inattention who completed the school-based screening but did not participate any further in the RCT (non-attending parents). The study also elicited the attitudes of teachers and other key school staff with regard to the acceptability of the interventions. Finally, the study sought to understand the feasibility of implementing such programmes in a community (school) setting.

## Methods

### Design

The study design of the PATCHWORK trial has been reported in detail elsewhere [16]. In brief, 12 schools across the East Midlands, UK were randomly allocated into one of three arms:

1. a ‘parent only’ intervention arm, where parents were invited to take part in the parenting intervention;
2. a ‘parent-teacher’ arm, where additionally teachers received a 1.5 hours session outlining the utility of ‘1-2-3 Magic’ in the home and classroom, an understanding of children’s needs and possible causes and functions of their behaviour, and encouragement to reflect on their current practice. Teachers also received weekly handouts summarising the information parents received during each week of the parenting intervention;
3. a ‘control’ arm, which did not receive the interventions until after final outcome data completion at 6-months follow-up.

All parents of children in Reception to Year 3 classes (aged 4–8 years) were asked to complete the Strengths and Difficulties Questionnaire (SDQ) [20] and those whose children scored ≥6 on its hyperactivity/inattention domain (representing top 20 % of population) were invited to take part in the main trial. Each parenting group was led by a group leader and facilitator.

This study is a nested qualitative process evaluation of the PATCHWORK RCT, to assess the feasibility and acceptability of delivering a group-based parenting intervention in a school setting for parents of children with high levels of hyperactivity/inattention.

#### Interviews

The semi-structured interviews were guided by separate parent and teacher interview schedules influenced by the process of hierarchical focusing [21], which included consideration of all aspects of the trial (screening, recruitment, the intervention [parenting group and teacher training/updates], and follow-up). Interviews were carried out by four authors (AV, KBA, JT and ES) following completion of the 6-month follow-up in the main RCT. The interview schedules were piloted in the initial interviews (parent and teacher), to assess their suitability before using across all schools and minor amendments made as necessary.

### Participants

A total of 22 primary caregivers and 29 teaching staff took part in the interviews, the sample comprised a range of socio-economic status (SES; determined by home/school postcode). Further demographic details are provided in Table 1. There were no significant differences in terms of gender, age of the child or baseline SDQ scores between parents who took part in the interviews and those who declined.

**Table 1:** Participant characteristics

Characteristic	Parents *n (%)*		Teachers *n (%)*	
*Gender*				
Female	21 (95 %)		23 (79 %)	
Male	1 (5 %)		6 (21 %)	
*Race*				
White British	21 (95 %)		Information not obtained	
Minority ethnic group	1 (5 %)			
*Relationship to child*	Mother	20	Class teacher	23^a^
	Father	1	Head/Deputy Head	4
	Grandmother	1	Head of Year	1
			Teaching Assistant	1
*Group*	Attending	8	Parent/teacher	18
	Non-attending	5	Parent only	10
	Control	9	Control	1

^a^Two of whom were also Special Educational Needs Co-ordinator [SENCO]

#### Parent recruitment

All parents, regardless of subsequent participation in the study, whose child scored ≥6 on the hyperactivity/inattention domain of the SDQ with an overall impairment score ≥ 2 on the SDQ (indicating significant clinical impairment) were invited to participate in the qualitative element of the study (*n* = 78). This cut-off represents children with borderline or elevated hyperactivity/inattention scores along with associated functional impairment, which may reflect clinically significant problems [22].

#### Teaching staff recruitment

From the 12 schools which took part in the PATCHWORK study, staff from 8 schools engaged with the present implementation research. Schools had the opportunity to withdraw from the research at any point. Teachers from one school withdrew following PATCHWORK recruitment and were subsequently not invited to take part in the present implementation study, another three schools declined the invitation to take part in the implementation study; the reasons for this included lack of time (*n* = 1) and change/illness in school staff (*n* = 2). A total of 29 teaching staff from eight schools chose to take part in either a group (four groups) (*n* = 21) or individual (*n* = 7) interview. Both choices were given to accommodate different schools’ preferences.

### Analysis

**Fig. 1:**
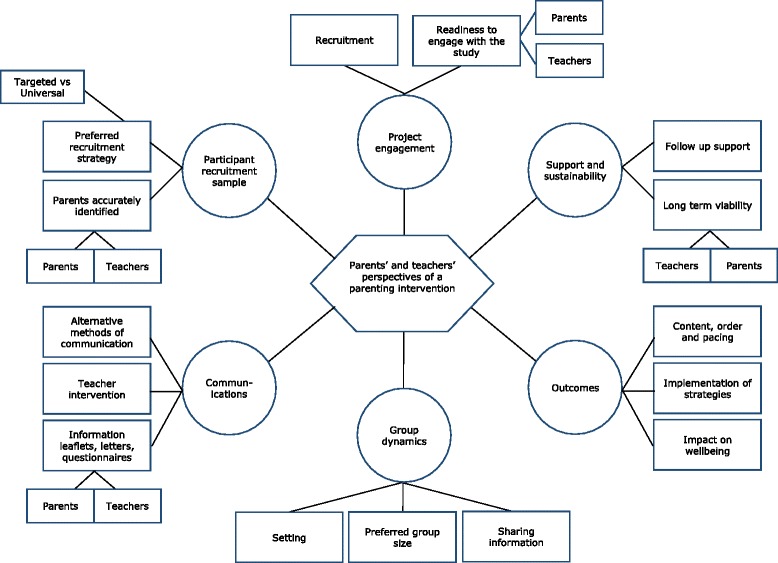
Thematic analysis network. Thematic analysis of parents’ and teachers’ views of a parenting intervention, showing the six main themes (final analysis)

All interviews were digitally recorded (with consent) and transcribed verbatim. As this was an explorative descriptive study, theoretical thematic analysis was used to identify, analyse and report patterns from the transcribed interviews using the guidelines of Braun and Clarke [23]. Analysis was not linear through the six stages, but rather a recursive process moving back and forwards through the stages, as needed to fully understand the data. The six stages followed were: 1) In order to familiarise ourselves with the data, one author (AV) listened to all interview recordings and checked the accuracy of the transcription. Three authors (AV, JT and ES) then read and actively re-read all transcripts, searching for meanings and patterns in the data. 2) Initial codes were generated by the aforementioned three authors who each put forward tentative coding categories derived inductively from one parent interview and one teacher group interview. 3) These codes were reviewed, refined and developed into a coding framework through discussion between the three authors. Once the themes had been defined and named, a preliminary thematic map was constructed and two authors (AV, JT) analysed the remaining interviews using this framework, making minor amendments to it following discussion and resolving disagreements through mutual consensus. 4) All data extracts corresponding to each theme were reviewed to ensure that the theme encompassed all data and all data were re-read to ensure that the themes were adequately defined. 5 & 6) A summary of all themes was written and the themes were defined and refined, including merging or renaming themes to ensure accurate representation of the data, alongside the broader existing literature (see Fig. 1).

Saturation of the data was reached prior to completion of analysis. The researchers’ perspective was that of critical realism, focusing on participant quotes as the material, whilst accepting that individual participants make meaning of their experience in light of their broader social context and that the researchers had knowledge about existing literature prior to analysis [23].

### Ethical approval

Ethical approval for the study was obtained from the University of Nottingham Faculty of Medicine Ethics Committee.

## Results and discussion

Inter-rater reliability of the interview transcript analysis was assessed by comparing a random sample of 20 % of quotes rated by the two primary raters: Kappa = 0.80, (*p* < .001), 95 % CI (0.71, 0.89).

A number of factors relevant to the implementation of parenting interventions were identified in the analysis which broadly fell into six themes: *Acceptability* - 1) Project engagement; 2) Participant recruitment; 3) Communications; and *Feasibility* - 4) Parenting group dynamics; 5) Outcomes; and 6) Support and sustainability (see Fig. 1). Each of the themes is discussed below. Quotes are used to illustrate each theme and to provide context from the participants’ viewpoint – parents’ and teachers’ perspectives are provided alongside each other and, where appropriate, differences in opinion are highlighted.

### Project engagement

#### Readiness to engage with the study

In terms of acceptability, most parents and senior teaching staff were able to discuss why they engaged with the research. Head/Deputy Head teachers reported that they took part in PATCHWORK because of being committed to research, their interest in behaviour management, and because they believed in the project’s focus on early intervention and providing support for parents. On the whole, teaching staff were not aware of what motivated the school to take part in the study and some reported a lack of consultation from senior teaching staff, which may have undermined the efficacy of the teacher-parent arm.

The reasons for parental engagement included altruistic reasons, such as wanting to help with the research, but were mainly because parents were experiencing difficulties with their child’s behaviour:

“I think for most people when they’ve got a child displaying these behaviours then they feel quite alone…So then when someone approaches you, you kind of feel a bit relieved…and you know some of these other people have got children with these issues as well” (attending parent).

In addition, some parents found that completing the questionnaires at both screening and follow-up was cathartic, “it is also a way to unload” (attending parent) or helped them to face up to the issues “they were good questions, they made me think about [name of child] and his behaviour” (control arm parent). No parents reported concerns with the screening process.

#### Recruitment

In order to address the issues of low uptake and high drop-out rates known in parenting interventions (see [10]), efforts were made in PATCHWORK to address potential barriers to attendance. For example, this included offering groups at a variety of times convenient to parents (daytime/evening/weekend), setting up stands at schools to talk to parents directly during screening recruitment, locating groups in the local community with parking, and providing childcare and refreshments.

Despite addressing these factors, recruitment levels were lower than previous parenting intervention research in families with a child with ADHD [5, 6]. This perhaps reflects the community (school) based recruitment as opposed to recruiting parents in a clinical setting. The response rate varied quite substantially across the 12 schools for both the screening questionnaire (14.3–46.0 %) and attendance at the parenting groups (30.8–53.8 %). Teachers confirmed that the attendance rates broadly reflected parental engagement with other activities within each school:

“Even parents’ evening we struggle to get them in, don’t we? Say out of about 27 children in your class, only 7 parents come” (Year 3/4 teacher).

Teachers cited a number of additional factors which they felt could have acted as barriers to parental engagement with the study including: stigma, lack of confidence, denial of the problem, literacy difficulties, work commitments, fear of the unknown, lack of responsibility/organisation, and apathy. Teachers reported many more potential barriers than parents, although parent interviews confirmed some of the same issues, particularly lack of confidence. For example,

“I think that is one of the things that put me off as I didn’t want to sit in a room full of people who I didn’t know. I’m just not that kind of person, I’m quite shy, so that’s why I didn’t go really” (non-attending parent).

Stigma has been highlighted in previous research as a key barrier to engagement relating to four areas: attending the group per se, the location of the group, the services delivering the programme and disclosing information in a group situation [24]. No parents raised the point that holding the group at school was a problem. A few parents described how being judged or labelled was a concern. In particular, one parent described her experience and the blame she felt for her child’s behaviour:

“Some of the reasons my daughter is the way she is, is because of the lifestyle that I led when she was younger. So I think it was more that I was worried about, not how my daughter would be perceived” (non-attending parent).

Contrary to previous research, most parents reported that stigma was not an issue. In fact, many parents referred to the positive link with the University of Nottingham, suggesting that the involvement of a University in delivering parenting interventions was helpful. However, teachers reported that certain parents are very wary of ‘officialdom’:

“[If] the paperwork’s got NHS as well on there, they’ll be, ‘is it going on their records?’ and ‘social services’, and they’ll go down that line of trail of thought” (Year 3 teacher).

This may explain why screening was low as our covering paperwork had various mandatory ‘official’ logos on it.

In the main trial, although 55.6 % of parents in the control arm were willing to complete questionnaires, attendance at the parenting intervention that was offered subsequently was low (23.6 %). This suggests a barrier not to engaging with research, but rather in attending the parenting intervention itself. One parent who fell into this category (participated in the research but did not attend the parent group) was interviewed. The barriers to attendance she reported were situational rather than psychological. Future research could explore this group of non-attenders further. Other non-participating parents and those who missed one or more sessions reported practical barriers (other prior commitments, working shifts), or having previously completed ‘1-2-3 Magic’ elsewhere as reasons why they did not attend the parenting group.

### Participant recruitment

The majority of parents were positive about being invited to take part in the study, although some parents questioned why they had been selected for the main trial:

“I think part of me, at times, wondered if I should have been part of the research because I was unclear whether my son’s behaviour was extreme enough to be part of it” (control arm parent).

Similarly, teaching staff questioned whether the majority of parents recruited were in need of additional support, as they felt many of the children did not display behavioural difficulties at school. As one teacher described, “You’ve had the ‘worried well’ responding which wouldn’t be our priority” (Head teacher). This suggests that the teacher believed that parents who engaged with the research were not those who were most in need of help, but those who responded to be reassured about their parenting skills. However, some teachers acknowledged that, “sometimes we don’t realise that the parent is having difficulties at home with their child” (Year 2 teacher). Consequently, it may be that parents did not perceive the intervention as relevant to them and this therefore may have acted as a perceived barrier [13].

Teachers felt that if the research team had worked more closely with teaching staff it would have increased the likelihood of identifying “those few who are not thriving at school and are causing problems at home” (Head teacher). In using a more targeted approach they felt that they could identify parents who “would’ve taken up that support, and would’ve benefitted” (Year 2 teacher). Teachers felt that they could recruit more parents than the research team alone, but this would have altered the study design from universal to targeted recruitment.

Previous research has found that parenting groups are often attended by parents with few risk factors and miss those with higher levels of needs [15]. It is possible that a more targeted approach to recruitment based on teacher nomination may have resulted in a different sample. However, the majority of parents were not in favour of teachers suggesting names of individuals or approaching parents in the screening/recruitment phase, instead preferring a universal strategy.

“[It] is better if the parents themselves actually volunteer to give the information, they might not have been happy about being put forward” (attending parent).

Arguably, universal access to parent programmes may increase acceptability and reduce the stigma associated with attending parenting groups [25]. However, such programmes are generally less effective than more targeted programmes [26]. The findings from the present study suggest that a combined approach to recruitment, whereby all parents are offered the opportunity to engage but parents of specific children with hyperactivity/inattention are additionally targeted by school staff may be acceptable to parents and teachers alike. As one parent described:

“I think that you could probably do it by [the research team] approaching the parents direct, but also having teachers reinforce the message” (control arm parent).

### Communications

#### Information leaflets and letters

Teachers’ knowledge of the project varied across schools. At some schools, staff felt that they were “kept very well informed” (Head teacher) but at others, teachers felt that communication could have been clearer. As one teacher expressed,

“It was you know you’ve got a letter to send out and we didn’t know anything about it really…we didn’t really know what was going to happen” (Year 2 teacher).

Written information about the project was given to teachers through teachers’ pigeon holes, but this was not always read:

“[Teachers] get thousands of pieces of paper in their pigeon holes and they very quickly go through and go ‘oh yes important, important, haven’t got time’” (Deputy Head teacher).

Teaching staff recommended that future research should include an information session with all teachers at the start of the project (although attempts had been made to do this in weekly staff meetings), and a designated teacher contact whom staff could approach with questions about the study. This may have helped address some of the barriers to communication with teachers, although not all could be overcome, due to the confidential nature of research, which teachers found difficult at times,

“I kind of find that [not knowing which parents took part in this study] a bit secretive and all a bit like, hush hush…on a need to know basis and I didn’t need to know” (Year 1 teacher).

Teachers were also keen to suggest ways in which communications to parents may have been improved to help recruitment. For example, they suggested making the initial letter “very straightforward” (Year 4 teacher) as its academic nature may have made some parents feel that “it’s not related to me” (Year 1 teacher). This is in line with the facilitators to attendance outlined by Koerting et al. [10]. Future research needs to balance the ethics committee requirements for detailed documentation and parents’ and teachers’ ability and time to read these documents.

#### Alternative methods of communication

Using alternative methods of communication may also help advertise programmes in future research. Some schools advocated the use of electronic media, such as email, texts, blogs, websites, school TV, and social networking sites to communicate with parents. Letters were seen as the least effective method of communication by teachers as they “often just seem to vanish on the way home” (Year 4 teacher). Parents reported that they benefitted from having text reminders, for example, about the parenting group or returning questionnaires. Alternative forms of communication may also help benefit parents with literacy difficulties [27].

#### Teacher session

One aim of the RCT involved providing teachers with information about the parenting strategies. Parents were keen that teachers were also informed about these strategies.

“I think it actually helps them do their job…if they get support and help with behaviour management across the board” (attending parent).

However, there was a general consensus from teachers that the content did not “really teach us anything new” (Year 3/4 teacher) but rather “complemented what we thought” (Year 3/4 teacher). In contrast, teachers in the other trial arms reported a desire to know more and gain knowledge about the ‘1-2-3 Magic’ programme.

“I’ve had parents come to me and ask for advice…and I think if we understood the programme then we could support parents” (Year 2 teacher).

As it was suggested that greater communication to all teachers would enhance the research, future research should engage with teachers face-to-face, prior to the screening stage.

### Parenting group dynamics

#### Sharing information with others

On the whole, parents did not inform teachers that they had participated in the parenting intervention. Some parents did share information with others, particularly the content of the programme with their partners. Although the group was open to both parents, it was not always practical for both to attend. Parents who had both attended expressed the usefulness of this.

“It was good for him [dad] to have an insight as to what goes off and how we can deal with things. He found it helpful too” (attending parent).

This parent went on to explain how sharing knowledge across family members meant that children “…can’t play us off (against each other)”. Previous research has shown that other family members can influence the decision to participate in family skills intervention programmes [28]. A study by Mockford and Barlow [29] found that discrepancies in parenting techniques between the attending parent attempting to change their approach and the non-attending parent could be a source of conflict. Future research should ensure that it is clear to all participants that the group is open to both parents or multiple care-givers.

#### Group size

As also identified by Smith et al. [14], some parents felt that a one-to-one session would be preferable to a group:

“I think it is ideal to do one-to-one, I think you can perhaps discuss more when it is one-to-one because, especially with it being parents that you don’t know, ‘cause I think that you are more afraid of opening up and revealing problems” (attending parent).

In contrast, one parent who was the only attendee would have preferred a group:

“It would have been nice if there had of been other parents there because it is quite nice…to meet other parents and chat about your children…just to make you think ‘I’m not the only one’” (attending parent).

Many parents who attended in a group discussed how they gained benefits from attending with others.

“It wasn’t intimidating or anything, you didn’t have to share anything if you didn’t want to. It was nice being a small group…nice to know that you are not alone” (attending parent).

Teaching staff suggested that having a staff member attend the group may be useful to help parents, “like a getting to know you” (Year 1 teacher).

#### Setting

At most schools, the parenting group was held within the school site but at some it was not always possible to do this. Teachers felt this may have been a barrier to attendance and believed that holding the group at the school may help increase attendance as it was somewhere where parents are familiar with, and it has “more validation…because they’d see it as part of school” (Year 1 teacher). Previous research suggests that although most parents are comfortable with parenting interventions in schools [30], some parents with bad memories of their own schooling or poor relationships with their child’s school may view the school location as a barrier [27]. Using buildings close to the school did not significantly influence uptake in the present study.

### Outcomes

#### Content, order and pacing of the group

The content, order and pacing of the group were generally favourably commented upon by attending parents. The step-by-step approach allowed parents to build on encouraging positive behaviours first before moving on to managing difficult behaviours. Even parents who professed to be already aware of the strategies discussed in the group, were able to tweak them to bring some success:

“I was quite impressed with some of the things that were suggested and although I was doing some of it, I wasn’t doing it to the extent that was discussed in the group…it’s actually worked. My way wasn’t quite the full way, it wasn’t working as well” (attending parent).

#### Implementation of strategies

Parents reported being initially sceptical of whether the strategies would work:

“Me and my husband were saying ‘oh I don’t know if it will work’ and not expecting great things but we’ll give it a go, but we were quite surprised at how quick things turned around once we’d implemented it” (attending parent).

Parents were aware that there was some slippage in the use of strategies:

“We have used it, I have to admit that we don’t always use it, but when it is practical and we do use it, it does work. We know what we should do; it is not always easy on a day-to-day basis” (attending parent).

Parental ‘motivation and capacity to change parenting practices’ has been noted as an ADHD-specific theme [14]. This was also supported in the present study and some parents gave up using strategies which worked initially but were not felt to be useful in the long-term.

“I suppose the daughter who has the behaviour problems has a very very short attention span and unless you sit with her in the timeout…you can take things away and you can give things back to her and it won’t make much difference either way. Some of them work for a very short period of time but none of them have worked for a consistent period of time” (attending parent).

#### Impact on wellbeing

As also found by Kane et al. [12] and considered important by Smith et al. [14], for some parents, the parenting group brought increased confidence in their parenting skills:

“I think that the positive stuff is already working and that has added to their [her children] life. And also it has been helping me…I think that it is making me feel more empowered” (control arm parent).

The current study suggests that the ‘1-2-3 Magic’-based programme was acceptable to parents of children with symptoms of hyperactivity/inattention. Given the positive evaluation of the content, future research should focus on addressing implementation issues rather than programme content, as also identified in other research [11]. This leads to discussion of the feasibility of the programme.

### Support and sustainability

#### Long term viability of the programme

The majority of schools were “quite happy for it [PATCHWORK] to carry on if it could” (Head teacher), although some reported that different strategies would be required to ensure that recruitment and uptake increased. One school described a successful parent programme in their school which had initially only been attended by motivated parents actively seeking help, but had become increasingly subscribed through word of mouth. This school advocated the use of parent champions, where “early adopters go back and spread the word that ‘yes, this is beneficial’” (Head teacher). This has also been suggested in previous research [10]. There was some evidence from the PATCHWORK study that parent champions could work. For example, one non-attending parent, who did not attend because of being the primary carer to two children with disabilities, learnt about the strategies from a parent who attended the group. She was very positive about the programme and its future viability:

“I have learnt a lot from the PATCHWORK…I hope that it does carry on in different schools and it is very helpful to other parents in the situation that I am in” (non attending parent).

It is also possible that the programme may continue to be sustained within the school environment, being run by staff members. One participating school has sent their SENCO on the ‘1-2-3 Magic’ training course and the programme continues to be run in this school, albeit using a more targeted method of recruitment.

#### Additional support

If the programme were to continue, suggested improvements included providing additional support. Some parents expressed that meeting after the programme had ended would be useful, “to find out how we were all going and just to refresh everybody on the key points of things” (attending parent). This view has also been expressed by participants in other behavioural management training programmes [31, 32]. Teaching staff suggested that it may be useful to continue to support parents through a support group on a social networking site where parents can “have a group together and then they can share ideas through that group as well” (Year 1 teacher).

## Conclusions

All parents who took part in the implementation interviews were positive about their involvement in the study. However, this must be considered in light of potential selection bias in that parents who benefitted may have been more likely to participate. Furthermore, the teaching staff process evaluation data were collected from 8 of the 12 schools, reflecting possible bias. Interviews were conducted by four authors, three of whom had been involved in the delivery of the parenting group. Positively, this may have helped disclosure in that parents and teachers had developed a rapport with the interviewers, but it may also have influenced non-disclosure of negative aspects. In addition, three of these researchers were involved in the interpretation of the data. The study was also limited by the relatively small sample size reflecting the low response rate from parents. Furthermore, the sample was restricted to parents of children aged 4–8 years from the first three year groups in Primary Schools in the UK. These factors must be borne in mind when considering the findings.

In terms of strengths, this study adopted a rigorous qualitative approach to provide insights into the desirability and acceptability of screening and offering a universal intervention rather than targeting a help-seeking population for ADHD-type problems. Multiple coders analysed the data and a high inter-rater reliability was achieved. The interview sample enabled the elicitation of teachers’ views about parenting programmes and collecting information from both attending and non-attending parents helped to highlight the issues around non-engagement and barriers to attendance. Data were collected from a range of schools (in terms of SES), staff in different roles and a mix of family members also ranging in SES. Furthermore, there were no significant differences in gender and age of child or baseline SDQ scores between those families that were interviewed and those which were not.

This study has shown that the acceptability of parenting interventions in the school environment was high. Both parents and teachers were enthusiastic about such groups and stressed the importance of parent/school collaboration in the organisation of these groups. The study adds to the body of evidence which demonstrates the difficulties in recruiting parents to parenting interventions [e.g. 11–15], even when practical barriers are considered and the group is held in a venue that the parent regularly attends. It was not clear in this study whether universal screening to select parents was beneficial. Although parents who responded reported positive aspects of completing the screening questionnaire, response rates were low in many schools. Engagement in the study was reflective of other aspects of parental engagement with their child’s school. Arguably, through completing screening, parents may be seeking help but may then not meet the study inclusion criteria; this may make parents less likely to seek help in the future [33]. Offering the group to all parents who perceive that they have difficulties with behaviour at home, regardless of the nature or severity of these difficulties may overcome this issue. Recruitment may be further enhanced by school staff inviting those parents whom they feel may particularly benefit from attending the group. Evidence from this study suggests that if this is done discretely, this may be acceptable to both parents and school staff.

The ‘123 Magic’-based programme was described favourably by attending parents, who largely seemed to benefit and had a lower than average drop-out rate compared to other parenting interventions. This may be because of the brevity of this programme, in comparison to other parenting interventions. Parents differed in their preferred group size, with some desiring one-to-one sessions and others favouring a larger group. The options for this in future research will depend on resources such as funding for staff and facilities (e.g. room size) within the school. The acceptability of the programme by non-attenders was influenced by factors such as fear of attending a group, already using ‘1-2-3 Magic’, work and other commitments. This qualitative evaluation has provided novel insights into the implementation of parenting programmes in the school environment for children at risk of ADHD. The findings have implications for future research, clinical practice and/or running parenting interventions in community (school) settings. Suggestions to improve the intervention arising from this study included: clearer communication, using basic language and technology to communicate with parents and teachers; offering booster and catch-up sessions for follow-up support; and greater collaboration with teachers, utilising teachers or parent champions to promote the programme effectively.

## Abbreviations

ADHD: Attention Deficit Hyperactivity Disorder
CLAHRC-NDL: Collaboration for Leadership in Applied Health Research and Care for Nottinghamshire, Derbyshire and Lincolnshire
NHS: National Health Service
NICE: National Institute for Health and Clinical Excellence
NIHR: National Institute for Health Research
PATCHWORK: PArents, Teachers and CHildren WORKing Together
RCT: Randomised controlled trial
SDQ: Strengths and Difficulties Questionnaire
SENCO: Special Educational Needs Co-ordinator
SES: Socio-economic status
